# γδ T Lymphocytes Coordinate Eosinophil Influx during Allergic Responses

**DOI:** 10.3389/fphar.2012.00200

**Published:** 2012-12-03

**Authors:** Maria Das Graças Muller de Oliveira Henriques, Carmen Penido

**Affiliations:** ^1^Department Pharmacology, Farmanguinhos, Oswaldo Cruz FoundationRio de Janeiro, Brazil

**Keywords:** integrin α_4_β_1_, selectins, MCP-1, IL-17, leukotriene B_4_

## Abstract

Tissue eosinophil infiltration, which is a hallmark of allergic and helminthic diseases, is mainly coordinated by T lymphocytes, via the production of eosinophilotactic chemokines. Among T lymphocyte subsets, lymphocytes expressing γδ T cell receptor have been determined as a key factor for eosinophil accumulation via direct and indirect mechanisms. This knowledge is strongly supported by the fact that, in different experimental models of eosinophilic airway inflammation and helminth-induced Th2 lung inflammation, an evident tissue accumulation of γδ T lymphocytes is observed. In addition, the depletion of γδ T lymphocytes is correlated with the impairment of eosinophil accumulation in inflamed tissue. γδ T lymphocytes are non-conventional T lymphocytes, which comprise a minor T lymphocyte subset, mainly distributed in the tissue, and present crucial roles in innate and acquired immune responses. γδ T lymphocytes recognize several danger- and pathogen-associated molecular pattern molecules and stress antigens in a MHC-independent fashion and can provide rapid tissue-specific responses, via the production of a wide range of chemical mediators capable to modulate other cell populations. These mediators include chemoattractant cytokines and chemokines that attract eosinophils into the tissue by either direct recognition (such as IL-5, CCL11/eotaxin), or indirect mechanisms via the modulation of αβ T lymphocytes and macrophages (through the production of interferon-γ, IL-4, and CCL2/Monocyte chemoattractant protein-1, MCP-1, for example). The present review presents an overview of how γδ T lymphocytes coordinate eosinophil accumulation in allergy, by focusing on their role in airway inflammation and by discussing the involvement of cytokines and chemokines in this phenomenon.

## Introduction

Eosinophils are major effector cells involved in allergy. They are found in increased numbers in peripheral blood, sputum, bronchial biopsies, and bronchoalveolar lavage (BAL) fluid of patients with asthma, as well as of experimental animals submitted to allergic challenge. Eosinophils produce a wide range of inflammatory mediators and granule enzymes, capable to cause tissue damage. Tissue eosinophilia during the allergic process is mainly coordinated by interleukin (IL)-5 and the CC chemokine CCL11/eotaxin both in experimental animals and human subjects (Beasley et al., 1989; Collins et al., 1995; Humbles et al., 1997; Robinson et al., 1999; Larangeira et al., 2001; Penido et al., 2001; Menzies-Gow et al., 2002, 2003; Kay and Klion, 2004; Phipps et al., 2004; Kay, 2005). Allergic airway inflammation is a T cell dependent phenomenon, characterized by a T helper (Th)2-driven immune response. Pre-clinical and clinical studies have defined that the Th2 subpopulation of CD4^+^ αβ T lymphocytes and their products (the Th2 cytokines IL-4 and IL-5) orchestrate the activation and the influx of eosinophils into the tissue during allergic inflammation (Corrigan et al., 1995; Gonzalo et al., 1996; Mazzarella et al., 2000; Moriwaki et al., 2011). However, there are solid evidences that the recruitment of eosinophils to the allergic site is coordinated by T lymphocytes bearing both T cell receptors (TCRs): the conventional αβ and the unconventional γδ T lymphocytes, as it will be further discussed.

## Distribution of γδ T Lymphocytes and Its Relevance in Physiopathology

γδ T lymphocytes comprise around 10% of T lymphocyte population in secondary lymphoid tissue and peripheral blood in rodents and humans (Groh et al., 1989; Hayday, 2000). Differently from the conventional T lymphocytes expressing αβ TCR, γδ T lymphocytes are abundantly distributed in epithelial (including the airway epithelium) and mucosal tissue (such as the intestine and lamina propria), comprising the intraepithelial lymphocytes (IEL; Hayday, 2000); albeit they are also present in the pleural cavity, which is a non-mucosal tissue (Penido et al., 1997). γδ T lymphocytes present innate cell-like features that permit their early activation following recognition of several antigens, such as pathogen-associated molecular patterns (PAMPS) and stress-induced ligands, such as damage-associated molecular pattern molecules (DAMPS; Bonneville et al., 2010). Via their TCRs, γδ T lymphocytes recognize stress-induced molecules expressed by cancer or infected cells, such as CD1c, T10, and T22. In addition, these cells express different receptors capable to recognize antigens, including: (i) Natural killer group 2 member D (NKG2D), which recognizes stress-related molecules, such as MHC class I-related chain A (MICA), MHC class I-related chain B (MICB), UL16-binding proteins (ULBPs), and retinoic acid early transcript-1 (RAE-1); (ii) dectin-1, which recognizes *β-glucans present in fungal cell walls*, and (iii) Toll-like receptors, which recognize PAMPS (Hayday, 2009). In response to antigen recognition, γδ T lymphocytes rapidly proliferate, produce cytokines, and present high cytotoxic activities, regulating, in a non-redundant manner, several physiopathological conditions. Compelling data have demonstrated that γδ T lymphocytes accumulate in sites of infection- and non-infection-associated types of inflammation. Interestingly, even though a major role for these cells has been described in host defense against pathogens and cancer, mainly due to their high capacity to display cytotoxic effects (Girardi and Hayday, 2005; Gomes et al., 2010) and to the fact it they are major producers of the Th1 cytokine, interferon (IFN)-γ, they have been shown to accumulate in sites of allergic inflammation (Hayday, 2000; Carding and Egan, 2002; Hahn et al., 2003; Penido et al., 2008).

## γδ T Lymphocytes in Allergy

γδ T lymphocyte accumulation during allergic inflammation is highly coordinated by the CCR2/CCL2 (Monocyte chemoattractant protein-1, MCP-1) pathway, even though a crucial role for the lipid mediator leukotriene B_4_ (LTB_4_) and its receptor (BLT1) has also been demonstrated (Costa et al., 2010). CCL2 and LTB_4_ are produced in tissue of allergic patients and experimental animals submitted to active sensitization and allergen challenge (Spinozzi et al., 1998; Yiamouyiannis et al., 1999; Schramm et al., 2000; Hahn et al., 2003; Svensson et al., 2003; Penido et al., 2008; Costa et al., 2009, 2010). When activated, at the *i*nflammatory site, these cells are capable to produce a multiplicity of cytokines and chemokines, with a unique plasticity to produce Th1, Th2, and Th17 cytokines, contributing to the development and regulation of several immune responses, including allergic inflammation. In addition to the important role of CCL2 and LTB_4_ in γδ T cell migration (Penido et al., 2008; Costa et al., 2010), an interesting paper from Kanehiro et al. (2001) highlights the role of TNF-α in γδ T lymphocyte activation during airway inflammation. These authors have shown that TNF-α-deficient mice submitted to a model of airway inflammation and hyperresponsiveness presented lower numbers of γδ T lymphocytes in the lungs, whereas γδ T cell numbers were increased in the lungs of TNF-α transgenic mice. It has been demonstrated that γδ T lymphocytes regulate airway responsiveness (Lahn et al., 1999) and that TNF-α presents an important role in early stimulation of γδ T cells in murine models of bacterial infection and LPS stimulation (Lahn et al., 1998). In this report, the authors show that, after sensitization and challenge, TNF-α deficient mice presented diminished levels of IL-5 and eosinophil counts in BAL fluid, as compared to wild type (WT) mice, even though γδ T cells presented a negative regulatory role in airway hyperresponsiveness.

Spinozzi et al. (1995) demonstrated that higher numbers of γδ T lymphocytes were found in BAL fluid of non-treated asthmatic subjects compared to non-atopic subjects. Interestingly, a large part of these cells (∼50%) expressed the CD30 molecule, a marker of T helper type 2 (Th2) cells (Romagnani et al., 1995; D’Elios et al., 1997; Fuchiwaki et al., 2011), produced high amounts of IL-4 (but not IFN-γ) and expanded *in vitro* under specific allergen stimulation, suggesting the existence and the local *in vivo* expansion of a Th2-type allergen-specific γδ T lymphocyte in the inflamed lungs of bronchial asthma patients, suggesting a role of these cells in local immune response to inhaled allergens.

The role of γδ T lymphocytes in allergy has been acclaimed to be complementary to the one of αβ T lymphocytes. It has been proposed that, since γδ T lymphocytes rapidly respond to the presence of antigen, they mount a prompt response, before αβ T cell immune response has been fully established (Christmas, 1992). Data obtained by Watkins et al. (1996) contribute to support this notion. They used a murine model of Th2 lung inflammation induced by infection with *Nippostrongylus brasiliensis* helminths, which are carried through circulation to the lungs, where they penetrate the alveoli and induce an inflammatory response that mimics the one developed during asthma in several aspects (Egwang et al., 1984; Ramaswamy et al., 1991; Ramaswamy and Befus, 1993a,b). These authors showed that the immune response was divided in an early and a late phase. The early phase of infection-induced response was characterized by intense influx to the airways of γδ (but not of αβ T lymphocytes); whereas the late phase was mainly characterized by CD4^+^ αβ T cells. Interestingly, the early phase coincided with the influx of neutrophils, whereas the late phase was characterized by the intense accumulation of eosinophils (in BAL and lung tissue). The role of neutrophils in allergic inflammation is still nuclear and considered minor to allergic response, even though significant neutrophil accumulation has been shown to occur during the acute phase of allergic response in different experimental models. Allergic neutrophilia might be important for eosinophil infiltration and has also been correlated with corticosteroid resistance in severe asthma, a phenomenon that might involve Th17 T cells (Montefort et al., 1994; Teran et al., 1995; Larangeira et al., 2001; Nakagome et al., 2012). γδ T lymphocytes can be correlated to neutrophil influx. Indeed, *in vitro* incubation of human circulating blood, γδ T lymphocytes with phosphoantigens (microbe derivatives of non-peptidic pyrophosphorylated molecules that powerfully stimulate these cells) induce the release of chemokine CCL8/MCP-2, which triggers neutrophil chemotaxis and activation (degranulation and secretion of the antimicrobial peptide defensin; Agrati et al., 2009). The regulation of neutrophil functions by γδ T lymphocytes has also been demonstrated in (non-allergic) *in vivo* experimental models, in which the production of chemokines is a central determinant for this phenomenon. γδ TCR deficient mice show decreased neutrophil accumulation in small intestine and lungs triggered by thermal injury or *Streptococcus pneumoniae* infection, due to the impairment of CCL4/MIP-1β, CXCL1 (keratinocyte derived chemokine, KC), and CXCL2/MIP-2 (Toth et al., 2004; Nakasone et al., 2007). However, the fact that there are few data in literature about the role of γδ T cells in neutrophil influx during airway inflammation lends support to the idea that eosinophil-, rather than neutrophil-induced migration, is coordinated by γδ T lymphocytes (Penido et al., 1997; Larsson et al., 2000; our unpublished data). In the late phase of the model of *N. brasiliensis* infection, the role of γδ T lymphocytes (whose numbers remained elevated in the BAL fluid of infected mice during the late phase) in the subsequent influx of eosinophils (and also of CD4 T cells) cannot be ruled out, as it will be further discussed. The parallel accumulation of γδ T lymphocytes and eosinophils in the tissue has also been demonstrated in another model of lung biased Th2 response: parasitic bronchitis triggered by the infection with *Dictyocaulus viviparus* (a bovine lung worm). Increased γδ T cell and eosinophil numbers were detected in the BAL fluid of infected calves, indicating the role of γδ T cells in the pathogenesis of *D. viviparus*-induced Th2 response and possible in eosinophil accumulation (Hagberg et al., 2005). An interesting piece of information that must be highlighted from this paper is that eosinophils recovered from BAL fluid of *N*. *brasiliensis-*infected mice were capable to produced eicosanoids involved in asthma upon *in vitro* (re)stimulation with calcium ionophore. Among those eicosanoids is LTB_4_, which has been shown by our group to be an important chemoattractant mediator to γδ T lymphocytes during allergy (Costa et al., 2010). Even though macrophages have been shown to be crucial cells for γδ T cell accumulation during several immune responses including allergy (Penido et al., 2003, 2008), this piece of data brings to light a possible mechanism of reciprocal cooperation between γδ T cells and eosinophils, which would be interesting to be thoroughly investigated.

### γδ T lymphocyte coordinate eosinophil influx: Evidences from experimental animal models of allergy

Nowadays, even though γδ T lymphocytes have been shown to accumulate in the airways of allergic human subjects and of experimental animals submitted to antigenic challenge, little attention has been given to the role of these unconventional T cells in allergy (Pawankar et al., 1996; Spinozzi et al., 1996, 1998; Schramm et al., 2000; Cui et al., 2003; Penido et al., 2008). However, the role of γδ T lymphocytes in the allergic reaction can be extended to their ability to coordinate eosinophil influx. A breakthrough paper published in 1998 by Zuany-Amorim et al. (1998) was the first one to show that γδ T lymphocytes are indeed required for the development of eosinophilic allergic inflammation. By using a model of lung allergic inflammation induced by intranasal OVA challenge in previously immunized mice, the authors showed that γδ knockout (KO) mice (γ^−^/δ^−^) presented an impaired Th2 response, as compared to WT mice from the same background. The absence of γδ T lymphocytes decreased specific immunoglobulin titers (anti-OVA IgE and IgG1) in serum, IL-5 and IFN-γ in BAL fluid, and eosinophil counts in bronchial tissue, as compared to WT-challenged mice. Overall, this paper clearly demonstrates that γδ T lymphocytes are essential for the initiation of Th2 responses *in vivo*, since the administration of recombinant murine IL-4 restored the ability of γδ KO mice to produce specific IgE and IgG1, to secrete IL-5, and to promote eosinophil influx in the lungs (BAL fluid). The release of IL-5 and subsequent tissue eosinophilia in the airways during allergic inflammation induced by OVA is largely dependent on the early IL-4. It has been demonstrated that γδ T lymphocytes can produce IL-4 (Ferrick et al., 1995; Vicari et al., 1996; Costa et al., 2009); however, the major cell source of such cytokine in this model of lung allergic inflammation has not been determined, since αβ T lymphocytes also secrete IL-4, and their numbers were decreased in the lungs of γδ KO mice. Indeed, it is noteworthy that γδ KO mice presented diminished counts of CD4 and CD8 T lymphocytes after challenge. Additional data in literature have demonstrated that γδ T lymphocytes can regulate eosinophilic airway inflammation. By using a model of 7-day repeated daily exposure of immunized to aerosolized OVA mice, Korsgren et al. (1999) demonstrated that γδ KO mice presented diminished eosinophil numbers in lung tissue as compared to WT matches. However, in this model, NK1.1^+^ cells seemed to be more important for eosinophil accumulation, which, by any means, reinforces the role of cells of the innate response in the regulation of allergic inflammation.

A report from Svensson et al. (2003) further investigated the mechanisms by which γδ T lymphocytes contribute to allergic eosinophil accumulation in the airways. By using a murine model of active sensitization and repeated aerosol challenge, these authors proposed that the contribution of γδ T lymphocytes to eosinophil accumulation during allergic airway inflammation occurs via an indirect mechanism, independent from the classical Th2 pathway. Their data demonstrated that, even though challenged γδ KO mice presented impaired IgE response, they were capable to mount normal IgG response, as well as expressed IL-4 in lung tissue in the same levels of challenged WT mice. The results presented in this paper suggest that eosinophil influx in OVA challenged mice was mediated by B lymphocytes, due to the fact that the number of B cells in the airways of mice lacking γδ T cells was reduced and that eosinophil accumulation was impaired in the airways of B cell KO mice submitted to antigenic challenge (Svensson et al., 2003). It is evident in literature that the development of allergic eosinophilia is accompanied by the local production of IL-4 and the shift toward Th2 predominant profile. It is well established that γδ T lymphocytes are especially important for the rise of Th2 responses via IL-4 production and by driving immunoglobulin isotype switching (Wen et al., 1994). It is known that IL-4 substantially induces the production of IgG1, whereas impairs IgG2b production. In this report, Svensson et al. (2003) have elucidated that γδ T lymphocytes do not contribute to IL-4-induced switch, albeit they present a role in systemic IgE response during allergic airway inflammation in mice (mainly during the sensitization phase, in which γδ T cells promote the specific IgE response and, subsequently after the challenge, γδ T lymphocytes trigger B cell accumulation in the airways). Therefore, γδ KO mice seem to be capable to develop and maintain Th2 response, suggesting that γδ T lymphocytes do contribute to the development of allergic airway inflammation in Th2-independent manner, via a different mechanism from αβ T lymphocytes, which is in accordance with Wang and HayGlass’s (2000) report. In contrast, a different set of data suggests that γδ T lymphocytes contribute to airway eosinophilia by enhancing Th2 response. McMenamin et al. (1994, 1995) showed that, by using a model of adoptive transfer of γδ T lymphocytes from OVA-tolerized mice into recipient normal mice, the suppression of Th2-dependent IgE antibody production occurred (without modifying parallel IgG responses). The *in vitro* challenge of these γδ T cells triggered the production of IFN-γ, which provides a potential mechanism for the inhibition of Th2 cell activation, suggesting that γδ T lymphocytes might modulate CD4^+^ αβ T lymphocyte functions in this experimental model. However, data published by Isogai et al. (2003, 2007) have demonstrated that a subpopulation of rat lymph node CD8^+^ γδ T lymphocytes, which produce IFN-γ, when intraperitoneally transferred into sensitized rats, impaired eosinophil accumulation in BAL fluid of recipient rats after aerosolized OVA challenge (when compared to control challenged rats). CD8^+^ γδ T lymphocyte recipient rats showed diminished levels of the Th2 cytokines IL-4, IL-5, and IL-13 in BAL fluid after challenge, whereas IFN-γ levels were higher than that in sensitized and challenge rat BAL fluid. Worthy of note, the adoptive transfer of IFN-γ-depleted CD8^+^ γδ T lymphocytes restored IL-4, IL-5, and eosinophil accumulation in challenged mice. These results indicate that these cells inhibit late airway responses and airway eosinophilia through the secretion of IFN-γ and that different γδ T cell populations can present divergent roles in allergic response.

This set of data also contrasts with the ones obtained by several other groups (Ferrick et al., 1995; Zuany-Amorim et al., 1998; Schramm et al., 2000; Costa et al., 2010), which clearly demonstrates that γδ T lymphocytes are capable to trigger Th2 response via the induction of Th2 cytokine production (including IL-4 and IL-5). This contrasting evidence might be explained by the differences in experimental models used in these studies (including sensitization and challenge protocols). A more relevant explanation might rely on the fact that γδ T lymphocytes are not a homogeneous population of lymphocytes, but are composed of different subsets, which can display opposite roles during the same immune response. Therefore, whether they display pro- or anti-inflammatory profiles can be determined by the γδ T lymphocyte subset involved in the immune response, as well as by its tissue distribution. Such statement has been well evidenced by previous works from O’Brien and Born groups (Huber et al., 2000; Cui et al., 2003; Hahn et al., 2003, 2004), as it will be discussed below.

## γδ T Subsets in Allergy

γδ T lymphocyte development within the thymus generates different γδ T cell subsets, bearing different TCR variable regions of either γ or δ chains, which emigrate in waves from murine thymus and populate different organs and tissues, in which they encounter specific antigens (Carding and Egan, 2002; Hayday and Pennington, 2007; Pang et al., 2012). These differences in the expression of variable regions can determine differences in γδ T lymphocyte tissue colonization and functions in the periphery. Among murine γδ T lymphocyte subsets, according to the nomenclature from Heilig and Tonegawa (1986), there are: Vγ1, mostly present in blood and secondary lymphoid organs; Vγ4, found in secondary lymphoid organs and in the lungs; Vγ5, which populate the epidermis; Vγ6, which colonize the uterine epithelium; and Vγ7, which are predominantly found at the small intestine epithelium (Bonneville et al., 2010). Among these subsets, Vγ4 and Vγ1 have been the major subsets studied in allergic processes. The importance of studying Vγ4^+^ T lymphocytes during airway allergic inflammation relies on the fact that these cells consist of a resident population in the upper respiratory tract and in the lungs (even though they are also present in other tissues) and is a subset that preferentially increases in the airways after antigenic challenge (Born et al., 2000; Carding and Egan, 2002; Hahn et al., 2003; Kim et al., 2008). As stated earlier, Vγ1^+^ T lymphocytes are present in blood, spleen, and lymph nodes, being also reported to be found in the small intestine and lungs (Pereira et al., 1995; Carding and Egan, 2002; Hahn et al., 2004). Vγ1^+^ T lymphocytes have also been shown to play a role in the aggravation of allergic response in the airways (Hahn et al., 2004). The roles of γδ T lymphocytes during the allergic response are multiple.

The role of γδ T lymphocytes in allergic response of the upper respiratory tract, such as in allergic rhinitis, has been briefly discussed in literature. It has been shown that around 25% of resident nasal T lymphocytes express γδ TCR, predominantly Vγ4/Vδ1 (Kim et al., 2008), suggesting a role for these cells in homeostasis and in immune responses of nasal mucosa. Indeed, other evidences suggest that γδ T lymphocytes play a regulatory role in mucosal immunity. Data from Russano et al. (2006) demonstrate that cloned γδ T lymphocytes recovered from nasal mucosa of patients with rhinitis and nasal polyposis (but not from non-allergic subjects), mainly of which are reported to be Vδ1^+^/CD4^+^ cells, recognized phospholipids extracted from pollen of different species. According to the fact that human γδ T lymphocytes recognize phospholipids via CD1 receptors (Spada et al., 2000), nasal mucosa cell from allergic patients recognized pollen-derived phospholipids, which induced γδ T cell activation (increased proliferation, IL-2 and IL-4 production, as well as IgE production). However, even though T lymphocytes bearing Vδ1, Vδ2, Vγ9, and Vγ4 chains were found in the blood of patients with perennial allergic rhinitis, no differences were found between the number of γδ T lymphocyte in rhinitis patients and control subjects (Sade et al., 2003).

**Figure 1:**
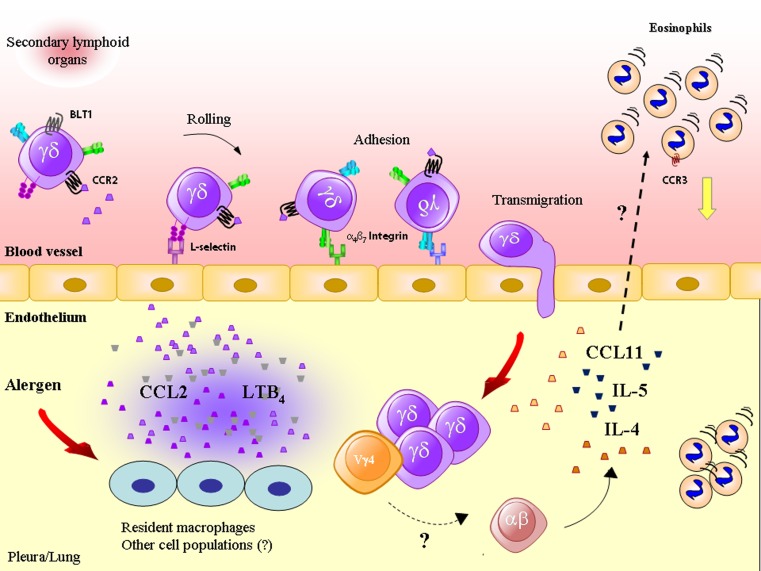
**Antigenic challenge triggers the production of CCL2/MCP-1 and leukotriene (LT)B_4_, which attract γδ T lymphocytes (including the Vγ4 T subset) into inflamed tissue through a mechanism dependent on adhesion molecules, such as L-selectin and α^4^β^7^ integrin**. γδ T lymphocytes produce cytokines and chemokines, such as IL-4, IL-5, and CCL11/eotaxin, important for eosinophil accumulation, a phenomenon that may also rely on the cooperation of γδ with αβ T lymphocytes.

Different γδ T cell subtypes can present diverse effects during immune responses, often in opposition. Compiled data in literature have clearly shown that γδ T lymphocytes contribute to the development of IgE-mediated responses, enhancing eosinophil tissue accumulation and airway hyperresponsiveness (Zuany-Amorim et al., 1998; Lahn et al., 1999; Schramm et al., 2000; Svensson et al., 2003; Costa et al., 2010), whereas other reports have demonstrated that γδ T lymphocytes also suppress airway hyperresponsiveness and IgE production during *in vivo* models of allergic lung inflammation (McMenamin et al., 1994; Lahn et al., 1999). According to this notion that different subpopulations of γδ T lymphocytes play heterogeneous functions, a work from Seymour et al. (1998) demonstrates that a subset of CD8^+^ γδ T cells presented no effect on the suppression of IgE production during a murine model of aerosolized OVA-induced unresponsiveness; however, the role of this subpopulation on the development of the allergic response was not investigated in this report. Indeed, the nature of these opposing roles of γδ T lymphocytes can in part be explained by the specific roles played by each γδ T lymphocyte subset. Those disparities in γδ T cell roles have been elegantly dissected in a study by Hahn et al. (2004). In this report, the authors demonstrate, by using a murine model of lung allergic inflammation induced by active intraperitoneal sensitization with OVA followed by airway challenge triggered by a 3-day repeated nebulization, that Vγ1^+^ T lymphocytes promoted allergic inflammation, whereas Vγ4^+^ T lymphocytes suppressed the allergic response. The depletion of Vγ1^+^ T lymphocytes before sensitization (but not before challenge) attenuated airway hyperresponsiveness (in C57BL/6 and BALB/c mice) and eosinophil numbers in BAL fluid of C57BL/6 mice (but not of BALB/c mice). When adoptively transferred into TCR δ chain KO mice submitted to allergic challenge, Vγ1^+^ T lymphocytes augmented the production of the Th2 cytokines IL-5 and IL-13, as well as increased the numbers of eosinophils in BAL fluid of recipient mice. It is important to note that, in this experimental model, Vγ4^+^ T lymphocytes presented a major role in the impairment of airway hyperresponsiveness, observed by the selective depletion of this subset before challenge (but not before sensitization), as well as by the adoptive transfer of this cells into TCR δ chain KO mice (Hahn et al., 2003, 2004). However, it is important to note that no changes in eosinophil counts in the BAL fluid were observed in challenged mice previously depleted of Vγ4^+^ T lymphocytes or in those that received Vγ4^+^ T lymphocytes by adoptive transfer (Hahn et al., 2003, 2004). However, in another work by the same group, it has been demonstrated that the treatment of mice with aerosolized antibodies targeting γδ T lymphocytes (either Vγ1^+^ and Vγ4^+^ T cells) impaired aerosolized intranasal OVA-induced eosinophilia in BAL fluid, suggesting that these cells might affect eosinophil trafficking during allergy (Lahn et al., 2004). However, it is noteworthy that mouse treatment with anti-Vγ1 and anti-Vγ4 antibodies also diminished the numbers of BAL macrophages. In addition to the knowledge obtained by this group of data that reveals divergent roles of different subsets of γδ T lymphocytes, it is noteworthy that these subsets also might be functionally important at different time points of the course of the immune response. It is important to highlight this observation due to the fact that data obtained from our group (Costa et al., 2009) show that the selective depletion of Vγ4^+^ T lymphocytes impaired eosinophil influx into inflamed pleura, in a model of pleural allergic inflammation, which is characterized by *in situ* production of IL-5 and CCL11 (Bozza et al., 1994; Larangeira et al., 2001; Penido et al., 2001, 2006). In addition to the fact stated above that Vγ4^+^ T cells comprise a major subset of γδ T cells in the lungs, Costa and colleagues showed that Vγ4^+^ T lymphocytes represented ∼40% of total γδ T lymphocytes in mouse pleura (being also found in peripheral lymph nodes). In accordance to a previous report from our group (Penido et al., 2008), that paper shows that the intra-pleural challenge with OVA in C57BL/6 mice triggered a marked eosinophil accumulation in inflamed pleura, which was preceded by the increase of γδ T lymphocytes. Among the γδ T cell populations that accumulated in the pleura of OVA challenged mice, Vγ4^+^ T cell numbers increased significantly (Costa et al., 2009) through a mechanism that so far seems to be mainly mediated by CCR2/CCL2 (but not by BLT1/LTB_4_; unpublished data; Figure 1). The migration of Vγ4^+^ T lymphocytes into inflamed tissue is in accordance with data in literature that demonstrate that this subset is found in lymphoid organs and peripheral blood, from which they migrate into inflamed airways (Carding and Egan, 2002; Hahn et al., 2003, 2004). It is, however, important to highlight that the resident γδ T lymphocyte population is likely to provide a further contribution to eosinophil tissue accumulation, as previously demonstrated in another model of pleural inflammation, triggered by the intra-pleural injection of bacterial endotoxin (Penido et al., 1997). In the model of allergic pleurisy, the specific depletion of Vγ4^+^ T lymphocytes by means of *in vivo* administration of monoclonal antibodies anti-Vγ4 TCR chain (produced by UC3 hybridoma) one day before sensitization (which was continued until challenge), markedly impaired, to the same level of non-challenged mice, the increase in eosinophil numbers in allergic pleura 48 h after OVA challenge. This result clearly demonstrates that Vγ4^+^ T cells present a crucial role in eosinophil influx during the model of allergic pleurisy. In this report, it is also shown that γδ T lymphocytes expressed intracellular IL-4, IL-5, and CCL11. After OVA challenge, the numbers of γδ T lymphocytes expressing intracellular CCL11 increased in the pleura of challenged mice, but there was only a tendency of increase in *in situ* IL-4^+^ and IL-5^+^ γδ T lymphocyte numbers. An important question to address is whether γδ T lymphocytes directly implicate eosinophil recruitment or indirectly modulate the production of IL-4 and of the eosinophilotactic mediators IL-5, CCL11, and CCL5 by other cell population, such as αβ T lymphocytes and macrophages. In another model of pleural inflammation triggered by LPS, it has been demonstrated that γδ T lymphocytes are crucial for tissue accumulation of eosinophils through a cross-talk with pleural macrophages (Penido et al., 1997). It is however noteworthy that LPS-induced eosinophil accumulation is not mediated by either IL-5 or CCL11 (Bozza et al., 1994; Penido et al., 2001), even though γδ T lymphocyte migration triggered either by LPS or OVA requires CCL2 and LTB_4_ (Penido et al., 2003, 2008; Costa et al., 2010). In addition to the well established role of Vγ4^+^ T lymphocytes in murine models of airway hyperresponsiveness (Hahn et al., 2003; Lahn et al., 2004), the role of these cells in eosinophil migration has also been demonstrated (Lahn et al., 2004; Costa et al., 2009), reinforcing the notion that Vγ4 T cell subset plays a crucial role in tissue eosinophilia during allergic inflammation.

It is important to briefly comment that Vγ4^+^ T cells are important producers of IL-17 and that the role of IL-17 in allergy has been an important focus of interest in immunopharmacology research. The role of IL-17^+^ γδ T lymphocytes (and of IL-17) in infection, tumor immunity, autoimmunity, and in the regulation of allergic airway inflammation has been reported (Shibata et al., 2007; Cornelissen et al., 2009; Murdoch and Lloyd, 2010; Ma et al., 2011). It has been demonstrated that IL-17 and IL-17 mRNA increase in the airways of asthmatic patients (Molet et al., 2001; Oboki et al., 2008). In regard to the participation of IL-17^+^ γδ T lymphocytes in airway inflammation, it has been recently demonstrated that those cells downmodulate central features of allergic reaction, including Th2 response and lung eosinophilia (Murdoch and Lloyd, 2010), and that these cells belong to the Vγ4 subset. Nevertheless, it is important to note that the acquisition of anti-inflammatory or regulatory functions by these cells might be stage dependent (as commented above), in addition to the fact that the inflammatory context differentially regulates the functions of these unconventional T lymphocytes (Carding and Egan, 2002). In a recent report from our group, we have described that a subpopulation of IL-17^+^ γδ T lymphocytes expressing chemokine receptors CCR6 (which characterizes IL-17 γδ producers; Haas et al., 2009) and CCR9 (the receptor of CCL25/TECK) migrate into the allergic pleura of OVA-immunized C57BL/6 mice coordinated by CCL25 and α_4_β_7_ integrin. It is noteworthy that, in this experimental model, CCL25 neutralization specifically impaired IL-17^+^ α_4_β_7_ integrin^+^ γδ T lymphocytes, but failed to alter the accumulation of other γδ or αβ T cell populations, as well as failed to impair eosinophil in the allergic site (Costa et al., 2012). This report reveals a particular *in vivo* migration pathway for IL-17^+^ γδ T lymphocytes, which requires CCL25/CCR9 axis and is mediated by α_4_β_7_ integrin. From this set of data, we can assume that IL-17^+^ γδ T lymphocytes might not be important for allergic eosinophilia; however, the role of IL-17 and IL-17^+^ γδ T lymphocytes in allergic responses is yet to be addressed. In addition, it is key to determine the existence of a corresponding population in allergic subjects, since IL-17-producing γδ T cells are still poorly characterized in human pathology.

## Concluding Remarks

Despite the fact that numerous studies of mouse models in combination with clinical studies have defined that Th2 CD4^+^ T lymphocytes are central cells to the pathogenesis of allergic inflammation in animal models and human subjects, overall data in literature exposed in the present review clearly evidences the crucial and non-redundant role of γδ T lymphocytes in allergic eosinophilia. However, the role of γδ T lymphocytes in eosinophil influx has recently received little attention, since only a few reports in literature bring this subject to light. Since substantial data demonstrate that γδ T lymphocytes together with αβ T lymphocytes are both crucial for eosinophil accumulation during allergy, it is critical to strengthen the importance of the study of γδ T cells in the pathogenesis of allergic diseases.

## Conflict of Interest Statement

The authors declare that the research was conducted in the absence of any commercial or financial relationships that could be construed as a potential conflict of interest.
